# Biological Drivers of Wilms Tumor Prognosis and Treatment

**DOI:** 10.3390/children5110145

**Published:** 2018-10-26

**Authors:** Hannah M. Phelps, Saara Kaviany, Scott C. Borinstein, Harold N. Lovvorn

**Affiliations:** 1 Vanderbilt University School of Medicine, Vanderbilt University, Nashville, TN 37232, USA; 2 Department of Pediatrics, Division of Pediatric Hematology-Oncology, Vanderbilt University Medical Center Nashville, TN 37232, USA; saara.kaviany@vumc.org (S.K.); scott.c.borinstein@vumc.org (S.C.B.); 3 Department of Pediatric Surgery, Vanderbilt University Medical Center Nashville, TN 37232, USA; harold.lovvorn@vumc.org

**Keywords:** Wilms tumor, nephroblastoma, tumor biology, biomarkers, therapy

## Abstract

Prior to the 1950s, survival from Wilms tumor (WT) was less than 10%. Today, a child diagnosed with WT has a greater than 90% chance of survival. These gains in survival rates from WT are attributed largely to improvements in multimodal therapy: Enhanced surgical techniques leading to decreased operative mortality, optimization of more effective chemotherapy regimens (specifically, dactinomycin and vincristine), and inclusion of radiation therapy in treatment protocols. More recent improvements in survival, however, can be attributed to a growing understanding of the molecular landscape of Wilms tumor. Particularly, identification of biologic markers portending poor prognosis has facilitated risk stratification to tailor therapy that achieves the best possible outcome with the least possible toxicity. The aim of this review is to (1) outline the specific biologic markers that have been associated with prognosis in WT and (2) provide an overview of the current use of biologic and other factors to stratify risk and assign treatment accordingly.

## 1. Introduction

Wilms tumor (WT), also called Wilms’ tumor or nephroblastoma, represents approximately 95% of childhood kidney tumors, with an incidence of approximately 7 cases per million children aged 15 years or younger [1]. WTs are classified among the embryonal tumors of childhood given the histologic and molecular resemblance to the embryonic kidney. Survival from WT now exceeds 90% at 5 years, in large part due to an increasing understanding of WT biology and the identification of molecular markers that associate with treatment resistance and poor prognosis. Decades of cooperative, multi-disciplinary trials have achieved these high overall survival rates, and research efforts are now aimed at minimizing toxic therapies while maintaining excellent survival. Additional research efforts have focused on the more lethal subsets of WT, including relapsed disease, anaplastic histology, and bilateral disease that collectively account for most treatment morbidity and cancer-related deaths. Wilms tumors historically were risk stratified by histology, defined as favorable (FHWT) or unfavorable histology (UHWT or diffusely anaplastic WT; 8%) [2].

Centralized research consortia have enabled large-scale analysis of this relatively rare disease. The two primary organizations from which treatment plans emerge are the Children’s Oncology Group (COG; formerly the NWTS) in North America and the International Society of Pediatric Oncology (SIOP) in Europe. Protocols established through both cooperative groups incorporate surgical resection of the tumor (either total or partial nephrectomy), chemotherapy, and radiotherapy when indicated. However, the timing and coordination of therapy differs between groups. COG protocols dictate upfront nephrectomy when possible, advocating earlier histologic diagnosis and more accurate staging information through lymph node assessment that together guide adjuvant therapy [1]. Upfront resection also provides an opportunity to minimize or avoid exposure to chemotherapy in certain subgroups. SIOP protocols, in contrast, recommend neoadjuvant chemotherapy without biopsy, followed by delayed resection. Adjuvant therapy is then tailored to histologic tumor features. This approach is associated with fewer cases of intra-operative tumor spill and lower post-operative tumor stage [3]. This review will focus on efforts by the COG, acknowledging that the work of each group is invaluable to the other.

Under COG protocols, WT stage is based on completeness of resection, perioperative rupture of tumor capsule, lymph node spread, and distant metastases (Table 1). Further risk stratification tailors therapy based on clinical (i.e., stage, patient age, tumor weight, and completeness of lung nodule response after six weeks of chemotherapy) and biologic (i.e., histology and loss of heterozygosity at 1p and 16q) features. Risk stratification according to histology and biology therefore helps to decrease exposure to toxic therapies for more favorable tumor features while maintaining excellent survival rates. The goal of this review is to summarize the key biological factors that impact current or potential future treatment regimens for WT.

## 2. Genomic and Molecular Alterations with Prognostic Significance in WT

### 2.1. Chromosome 11

Chromosome 11p harbors two distinct WT suppressor genes, *WT1* and *WT2*. Early steps to understand WT biology were driven by the observation that children with WAGR syndrome (Wilms tumor, Aniridia, Genitourinary Malformations, and Retardation) had a constitutional deletion of 11p13. The specific gene, *WT1*, was isolated by identifying the minimum deletion region of that locus [5]. *WT1* is a transcriptional regulator likely involved in coordinating differentiation of genitourinary tissues, though its exact function in kidney development remains incompletely characterized [6]. *WT1* mutations occur in only about 15% of sporadic WT, suggesting the involvement of other key genes in tumorigenesis. Loss of both copies of *WT1* has been described as a hallmark of WT development in WAGR and other constitutional genetic conditions, such as Denys-Drash and Frasier Syndromes, suggesting loss of tumor suppressor function.

A second gene, *WT2,* located at 11p15, is altered at a much higher frequency in WT (~70%) [3]. Linkage analysis has mapped the gene for Beckwith-Wiedemann Syndrome (BWS, another WT predisposition syndrome) to the 11p15 locus, and 11p15 is now recognized as a region of genomic imprinting. In normal development, the maternal allele for *IGF2* and the paternal allele for *H19* are silenced at this locus. Loss of imprinting (LOI) and subsequent activation of the normally silenced maternal *IGF2* (insulin-like growth factor 2) allele result in the most common genetic alteration in WT, occurring in 30–50% of sporadic WT [7,8]. Paternal uniparental disomy, whereby one chromosome is lost and the remaining chromosome is duplicated, may also lead to copy neutral loss of heterozygosity (LOH) at 11p15 and subsequent overexpression of *IGF2*. It is thought in the embryonic kidney that if biallelic methylation of 11p15 occurs before induction, high levels of *IGF2* cause preferential proliferation of the metanephric mesenchyme, impair development of the nephron, and yield persistence of these rogue progenitors in the form of nephrogenic rests (NR), the putative precursor lesion of WT. This event hypothetically results in intralobar NR. If the biallelic methylation of 11p15 occurs after induction, high levels of IGF2 prevent terminal epithelial differentiation, resulting in perilobar NR [9]. It is thought that intralobar NR occur at an earlier stage in renal development than perilobar NR. Notably, *WT1* alterations are also associated with development of intralobar NR [10]. As noted, NR represent the precursor lesion of WT, but a second genetic event within this population of stem cells likely initiates development of WT.

Importantly, the *WT1* mutation and 11p15 LOH or LOI are thought to drive distinct pathogenetic mechanisms for the development and/or progression of WT; however, these events are not necessarily independent given the close proximity of the 11p13 and 11p15 loci. In one study, all patients with *WT1* mutations also had 11p15 LOH, yet 11p15 LOH was identified without *WT1* mutations in a proportion of cases. Therefore, 11p15 is likely the more sensitive prognostic indicator [11].

### 2.2. Loss of Heterozygosity at 1p and 16q

Early studies utilizing LOH mapping identified chromosomes 1p and 16q as regions of interest in WT genetics, with 17% of tumors harboring LOH at 16q and 12% at 1p [12,13]. Concurrent LOH at both loci was associated with adverse prognosis among favorable histology WT (FHWT) [12]. Since then, multiple studies in North America and Europe have suggested that LOH at 1p and/or 16q associates with relapse and overall poor prognosis [14,15,16,17]. Most recently, the NWTS-5 clinical trial demonstrated that combined tumor-specific LOH of chromosomes 1p and 16q in patients with Stage III and IV disease was associated with inferior 4-year event-free survival (EFS) and overall survival (OS) in a cohort of greater than 1700 patients [18]. Based on these findings, the COG clinical trials AREN0532 and AREN0533 intensified chemotherapy for patients with FHWT that demonstrated LOH at 1p and 16q. To date, combined LOH at 1p/16q is the only molecular marker employed in risk stratification. Though it appears to be a sensitive marker for predicting relapse, LOH at 1p and 16q is not broadly specific, as this combination was present in only 9.4% of relapsed tumors. Overall, combined LOH at 1p and 16q is present in only 5% of FHWT [18].

Efforts to identify a mechanistic explanation for these observations demonstrated that 16q loss associated interestingly with LOI at 11p15. The authors speculated that haploinsufficiency of the CCCTC-Binding Factor (*CTCF*) gene, which maps to 16q22 and codes for an insulator protein that regulates *IGF2* imprinting, is the mechanism behind this association. Indeed expression of *CTCF* in tumors with LOH at 16q was half that of tumors with normal chromosome 16 [19].

### 2.3. Copy Number Gain at 1q

Copy number gain of chromosome 1q is a commonly observed genetic abnormality in WT and is present in approximately 30% of tumors [20]. After several smaller, retrospective studies suggested a correlation between 1q gain and tumor recurrence, the Children’s Cancer and Leukaemia Group, NWTS, and SIOP independently confirmed poorer EFS and OS in both pre-treated and untreated patients with 1q gain in larger cohorts [21,22,23,24,25,26]. Data collected through the NWTS-5 clinical trial was used to assess the prognostic significance of 1q gain in FHWT with sufficient power to detect survival differences within stage groups. Among all stages, 8-year EFS was 77% (95% CI, 72–81%) for patients with 1q gain, and OS was 88% (95% CI, 83–91%). For patients without 1q gain, 8-year EFS was 90% (95% CI, 88–92%), and OS was 96% (95% CI, 94–97%) [20]. No significant difference in histologic predominance based on presence or absence of 1q gain was observed. Stage IV disease was more common among patients with 1q gain compared to those without (18.3% vs. 9.4%, *p* < 0.001), suggesting that the abnormality is associated with a more aggressive and malignant phenotype. In a stage-by-stage comparison, EFS was significantly poorer for patients with 1q gain and Stage I, III, and IV disease [20]. 1q gain was not independent of 1p and 16q loss, which is not surprising given that isochromosome 1q and translocations between chromosomes 1 and 16 are common genetic abnormalities in WT. Both alterations can cause loss of 1p or 16q and gain of 1q [20]. It remains unclear which abnormality and which relevant gene(s) accounts for the poorer outcomes in patients with these genetic abnormalities. Of note, 1q gain has been associated with poor outcomes in other embryonal tumors including neuroblastoma, pediatric ependymoma, medulloblastoma, Ewing sarcoma, as well as other adult tumors [27,28,29,30].

### 2.4. Alterations at 17p

*TP53* mutations have been identified in 50–86% of anaplastic WT [31,32]. Presence of p53 in anaplastic regions but not in non-anaplastic regions of the same tumor suggests that anaplasia stems from a clonal event that takes place in a FHWT. A recent COG study using patient samples collected in NWTS along with the TARGET Data Matrix demonstrated *TP53* mutations in 48% of patients with diffuse anaplastic WT (DAWT) (*n* = 118) [31,33]. An additional 11% without mutations had loss of 17p13 (*TP53* locus). *TP53* abnormalities do not appear to associate with stage of DAWT but are associated with significantly worse disease-free and overall survival (OS) for patients with Stage III or IV DAWT [31]. The association between DAWT and *TP53* mutation has been substantiated in multiple other studies [32,34,35]. The authors speculated that the low degree of anaplasia in most tumors lacking *TP53* mutation and/or copy loss indicate that detection of a *TP53* abnormality reflects the “burden of anaplasia. The “burden of anaplasia” may not have a survival impact on Stage I and II anaplastic tumors if completely resected as compared to Stage III and IV anaplastic disease, which is at higher risk of residual or systemic tumor after surgery [31]. Another recent study sought to characterize *TP53* status in a cohort of fatal WT cases. *TP53* alterations (detected by sequencing, copy number, and/or protein expression) were detected in 52% (45/86) of fatal WT [35]. *TP53* alterations were detected in 97% (29/30) of tumors with DAWT but were also present in 26% of non-anaplastic tumors. Of note, many of the non-anaplastic tumors partially fulfilled anaplastic criteria (e.g., nuclear unrest). These results, coupled with the observation that p53 positivity on IHC was not limited to anaplastic cells, but expanded areas surrounding anaplasia, suggest that *TP53* status could also be a screening tool for tumors with partial phenotypes [35,36].

### 2.5. Loss of Heterozygosity at 4q and 14q

In addition to alterations at the *TP53* locus, molecular profiling has demonstrated significant associations between anaplastic histology and loss of 4q and 14q [34]. Specific candidate genes involved in WT pathogenesis at these latter loci have not been identified yet, and the significance of these genomic alterations remains unknown.

### 2.6. MYCN Amplification

Genomic amplification of the *MYCN* gene has repeatedly been described in WT as well as other embryonal tumors, most commonly in neuroblastoma [37,38,39,40]. Overexpression of *MYCN* in WT has been identified as a potentially prognostic feature [34,41,42]. Interestingly, *MYCN* gain was present in higher proportion (>30%) among a cohort of pre-treated anaplastic tumors compared with a parallel study analyzing a mixed cohort of anaplastic tumors (which included tumors that were not pre-treated). This suggests that *MYCN* gain could confer treatment resistance [34,43]. Notably, *MYCN* gain is not limited to anaplastic WT, and its association with poorer relapse-free and overall survival is independent of histology [42,44]. The P44L mutation has been identified as a potentially activating mutation leading to *MYCN* gain in WT [44].

### 2.7. LOH at 11q

Among a cohort of 225 tumors treated on the SIOP protocol, 19.6% (44) demonstrated allele loss on chromosome arm 11q. Frequency of LOH at 11q was 3–4 times higher among mixed type and diffuse anaplastic tumors compared to favorable histology tumors. Loss of the entire long arm of chromosome 11 was associated with higher rates of relapse and death [16]. Other studies have also demonstrated a correlation between LOH at 11q and anaplasia, tumor recurrence, and death, indicating that this region is likely prognostically relevent [15].

## 3. Current Risk Assignment and Treatment Strategies for Wilms Tumor

This next section will focus on how these biologic data have stratified the treatment approach to WT patients having specific clinical, histologic and molecular alterations. The overarching objective of these risk strata is to tailor therapy that is less toxic for more favorable WT cases and intensified for those patients having a higher risk of treatment failure.

### 3.1. Very Low Risk

Very low risk Wilms Tumor (VLRWT) is defined as age less than 2 years, favorable histology, small tumor (<550 g) confined to the renal capsule and completely resected. This definition first emerged out of NWTS-5 study after analyses showed that adjuvant chemotherapy did not improve survival in certain subsets of low risk patients [45]. The subsequent attempt to eliminate adjuvant chemotherapy among patients meeting very low risk criteria resulted in early termination due to EFS (86.5%) at the 3-year interim-analysis, which was inferior to the set stopping point (90%) [46]. However, later analysis revealed high salvage rates with three-drug chemotherapy (vincristine, dactinomycin, and doxorubicin) and site-specific radiotherapy, resulting in an excellent overall survival [47].

Using this definition for VLRWT, the subsequent COG Trial AREN0532 enrolled 116 patients for nephrectomy-only therapy. At four years, 12 patients had relapsed (4-year EFS 89.7%), and OS was 100%. Biologic features were evaluated as potential indicators of children at risk for relapse. 1q gain, 1p loss, and/or 16q loss and mutation of *WT1* were not associated with relapse; however, 11p15 methylation status, particularly LOH and LOI, was associated with relapse in this otherwise favorable group [48]. The authors concluded that the observation-only approach to VLRWT potentially evades chemotherapeutic drug toxicity and complications from central lines, yet exposes relapsed patients to anthracyclines and radiation, which may have been avoided if the standard adjuvant vincristine/dactinomycin had been delivered. While 11p15 status is a potential predictor of relapse, with a 20–25% risk of recurrence in patients with LOI/LOH compared to a 3% risk in those with no LOI/LOH, most patients with 11p15 methylation abnormalities are predicted not to relapse [48]. The *WT1* mutation has also been associated with relapse among VLRWT treated with nephrectomy-only and could be applied instead of or in addition to 11p15 methylation status to predict risk of relapse in these patients [11]. Based on the results of AREN0532, the current treatment strategy for VLRWT is nephrectomy followed by observation (Table 2) [48]. Given these encouraging findings, future studies are under consideration to increase the tumor weight limit and expand the age range included in the definition of VLRWT [48].

### 3.2. Low Risk

#### Stage I and II FHWT without LOH at 1p/16q

WT patients having Stage I or II disease and not meeting very low risk criteria are treated with upfront nephrectomy, when feasible, followed by adjuvant chemotherapy with vincristine and dactinomycin (Regimen EE-4A, Table 2). This low risk category has not been evaluated on a separate AREN trial to date, but NWTS-5 data showed that this group attained 4-year EFS and OS of 91.2% and 98.4%, respectively [18].

### 3.3. Standard Risk

#### 3.3.1. Stage I and II FHWT with LOH at 1p/16q

Prospective, large-scale analysis through NWTS-5 identified adverse prognosis among FHWT patients having LOH at 1p/16q. These data prompted intensification of therapy for patients with this combined genetic marker. Under AREN0532, patients with Stage I or II FHWT demonstrating concurrent LOH at 1p and 16q were provided with a standard risk treatment regimen in place of the low risk regimen used for Stage I and II FHWT without LOH 1p/16q. The standard risk regimen consists of upfront nephrectomy when possible followed by Regimen DD-4A (doxorubicin, vincristine, and dactinomycin) without radiation therapy (Table 2). EFS for this subset of patients was 83.9% (64.9–93.1%), and OS has not been published at this time [49].

#### 3.3.2. Stage III FHWT without LOH at 1p/16q

The current approach to treating Stage III FHWT, carried over from NTWS-5, includes upfront nephrectomy, when feasible, followed by Regimen DD-4A and abdominal radiation therapy (Table 2). The AREN0532 study demonstrated continued excellent EFS (88% [95% CI, 85–91%]) and OS (97% [95% CI, 95–99%]) for Stage III FHWT without combined LOH 1p/16 treated with this protocol [50]. The study additionally sought to identify clinical, pathologic, and molecular features associated with worse prognosis that might potentially be used for future risk stratification. Poorer EFS was associated with positive lymph node status and LOH at 1p or 16q. Patients with positive lymph nodes and LOH at 1p or 16q had EFS of only 74%. Gross residual disease and peritoneal implants were not associated with poorer EFS [50]. 1q gain status was not available for the AREN0532 cohort, but future therapeutic protocols under consideration include assessment of 1q gain in addition to LOH at 1p/16q so that doxorubicin might be omitted in patients that lack these risk factors [50].

#### 3.3.3. Stage IV FHWT with Isolated Lung Metastases Responding Completely to Chemotherapy but without LOH at 1p/16q

Under former NWTS protocols, all patients with metastatic lung disease received lung radiation. Recent changes in treatment protocols have focused on reducing the number of patients exposed to lung radiation without compromising EFS and OS. On AREN0533, the need for lung radiation in patients with Stage IV FHWT having isolated lung metastases was determined by LOH at 1p/16q as well as lung nodule response to chemotherapy (Table 2). Data from NWTS-5 demonstrated the association between combined LOH at 1p/16q and adverse prognosis, serving as the rationale to incorporate LOH at 1p/16q as a factor to stratify further risk among Stage IV FHWT [18]. The addition of lung nodule response to the risk stratification scheme for AREN0533 was derived from SIOP protocols, which dictate 6 weeks of pre-nephrectomy vincristine/dactinomycin/doxorubicin to determine next steps in therapy. After 6 weeks of treatment, if a complete response (as defined by chest computerized tomography) is achieved without evidence for remaining lung nodules, either from chemotherapy or surgical resection, lung radiation is excluded from the subsequent treatment regimen. With this strategy, the SIOP has reported 5-year EFS of 77% and OS of 87%, comparable to the 76% EFS reported by NWTS-5 [54,55].

Based on the protocol outlines in AREN0533 and the encouraging SIOP findings, currently, patients with Stage IV FHWT but without LOH at 1p/16q who demonstrate a complete response after 6 weeks of DD-4A therapy continue with Regimen DD-4A regimen and do not receive lung radiation. However, patients without a complete lung nodule response or with LOH at 1p/16q are transitioned to the higher risk stratum (see 3.3.1 below). Among 292 patients presenting with isolated lung metastases enrolled on AREN0533, 133 (46%) demonstrated complete response after 6 weeks of DD-4A. Four-year EFS was 79.5% (95% CI, 71.2–87.8%) and OS was 96.1% (95% CI, 92.1–100%) for patients without LOH 1p/16q and showing complete response. Initial lung radiation was avoided in approximately 40% of patients. A post hoc analysis of prognostic significance of 1q gain was performed, which associated significantly with poorer 4-year EFS and a trend toward poorer OS among patients demonstrating otherwise a complete pulmonary response to DD-4A [51]. This result suggests a role for assessing 1q gain in this stratum to identify patients who may not be good candidates for omission of lung radiation despite the resolution of lung nodules with chemotherapy.

### 3.4. Higher Risk

#### 3.4.1. Stage III or IV FHWT with LOH 1p/16q

Again, in response to poorer prognosis among patients with LOH 1p/16q established in NWTS-5, AREN0533 augmented therapy for patients with Stage III or IV FHWT and LOH 1p/16, assigning these cases to the intensified protocol defined as Regimen M (incorporating cyclophosphamide and etoposide to vincristine, dactinomycin, and doxorubicin) in addition to radiation therapy (Table 2). Preliminary results published in abstract form indicate that 4-year EFS for patients treated under this protocol was 91.5% (95% CI, 78.5–96.8%), which is significantly better than those historically treated with Regimen DD-4A [49].

#### 3.4.2. Stage IV FHWT without LOH at 1p/16q but with Isolated Lung Metastases Responding Incompletely to Chemotherapy

Patients without LOH 1p/16q and with isolated lung metastases initiated on DD-4A who do not exhibit complete pulmonary response are converted to the higher risk stratum to receive Regimen M. Four-year EFS and OS for patients with incomplete response was 88.5% (95% CI, 81.8–95.3%) and 95.4% (95% CI, 90.9–99.8%), respectively. No significant association was detected between 1q gain and EFS or OS in patients with incomplete response [51].

#### 3.4.3. Stage IV FHWT with Extrapulmonary Metastases

Stage IV FHWT with extrapulmonary metastases is currently treated with upfront nephrectomy, if feasible, followed by Regimen M and radiation therapy to the tumor bed and involved sites. Of 349 patients with Stage IV FHWT assessed for inclusion in AREN0533, 52 patients had extrapulmonary metastases. Outcomes for this group are yet to be described in detail.

### 3.5. High Risk

Tumors demonstrating diffuse anaplasia (DAWT) are defined as high risk. As previously described, several biologic markers, including *TP53* mutation, LOH at 4q and 14q, and LOH at 11q, have been shown to associate with DAWT. High risk, DAWT is currently treated with Regimen UH-1, which incorporates cyclophosphamide, carboplatin, etoposide, vincristine, and doxorubicin. Under AREN0321, patients with metastatic (Stage IV) DAWT also had the option to participate in a window study to assess the antitumor activity of vincristine and protracted-schedule irinotecan against metastatic (Stage IV) DAWT. The results of the phase II window study indicate preliminarily that among 14 participants with Stage IV DAWT, 11 had partial response and 3 had progressive disease with treatment. Patients with a partial response had vincristine and irinotecan incorporated into Regimen UH-1 (called Regimen UH-2). Notably, two of the three non-responders also did not respond to additional treatment [56]. Based on these results, continued monitoring of the efficacy of neoadjuvant vincristine and irinotecan for metastatic DAWT is underway. Among Stage II-IV DAWT treated with Regimen UH-1/UH-2, three-year EFS was 69% (95% CI, 56–80%), representing improved EFS from patients treated on Regimen I (lacking carboplatin) under NWTS. However, toxicity was also increased with Regimen UH-1/UH-2 as three patients (4.5%) died from treatment toxicity [52].

### 3.6. Bilateral, Multicentric, or Bilaterally-Predisposed Unilateral Wilms Tumor

#### 3.6.1. Bilateral WT

Bilateral Wilms Tumor has historically been associated with poor outcomes (4-year EFS 56% according to NWTS-5) [53]. The current regimen for treating bilateral WT is derived from AREN0534 with the primary goal of improving EFS and OS while preserving renal parenchyma. The protocol starts with intensive neoadjuvant chemotherapy (VAD Regimen; vincristine, dactinomycin, and doxorubicin) and mandates definitive surgery by 12 weeks. Adjuvant therapy is modified based on the histologic response. Among 242 patients enrolled on AREN0533 for bilateral WT, 21.1% had a genetic predisposition syndrome. Among 189 patients treated and evaluated, four-year EFS and OS were 82.1% (95% CI,73.5–90.8%) and 94.9% (95% CI, 90.1–99.7%), respectively. Definitive surgery was performed by 12 weeks in 84% of patients, of which 39% were successfully treated with bilateral nephron-sparing surgery [53]. Further studies are needed to assess the role of genetic markers in predicting risk of relapse.

#### 3.6.2. Genetically Predisposed WT and Diffuse Hyperplastic Perilobar Nephroblastomatosis (DHPLN)

Patients who develop WT in the context of a genetic predisposition syndrome (e.g., WAGR, Denys-Drash, BWS) are at high risk for metachronous or multicentric tumors through 8 years of age. Preservation of renal parenchyma is of utmost importance in this patient population. To this end, current treatment protocols involve 2-drug induction with vincristine/dactinomycin with the goal of facilitating partial, rather than complete, nephrectomy. Data collected under AREN0533 for this specific treatment strategy has not yet been published.

A similar chemotherapy regimen with vincristine/dactinomycin has been utilized for the goal of preventing development of WT from DHPLN. Results of this arm of AREN0533 have also not yet been published.

## 4. Conclusions

Significant progress has been made to identify indicators of poor prognosis with WT and to adjust treatment algorithms accordingly. So far, LOH at 1p/16q is the only biologic marker included in WT risk stratification, but emerging evidence has identified several additional markers of poor prognosis, including mutations of *WT1*, 1q gain, alterations at 17p, LOH at 4p and 14q, *MYCN* amplification, and LOH at 11q. Incorporation of additional biologic markers, particularly 1q gain, into treatment protocols can be expected in the coming years. As one example to reduce toxic exposures using tumor-specific biology moving forward, doxorubicin might be omitted in Stage III FHWT without LOH 1p/16q and 1q gain (see Section 3.3.2), although such a proposal would require evaluation through a rigorous cooperative trial to determine no change in adverse event frequency while mitigating early and late treatment side effects. Additional efforts are underway to identify differences in tumor genetics based on race and ethnicity, which could provide further insight into risk stratification and allow for even more personalized treatment strategies.

As risk stratification becomes more dependent on biological markers, it is important to consider the reliability of analytical tests and methods employed to identify these factors. WT are often huge at resection and show regions of histologic variability regarding cellular predominance (i.e., content of blastema, epithelia, and stroma vary) and necrosis. The degree to which a single, small sample (usually ~250 mg in size) can reliably represent an entire tumor has not been adequately explored in WT or other large, embryonal tumors. Recently, one study demonstrated that certain genetic markers thought to represent earlier events in tumor development, such as LOH at 11p15, are uniformly detected in WT specimens. However, later genetic events, such as 1q gain are variably detected in different samples derived from the same tumor. The authors estimated that at least 3 samples per tumor should be analyzed to detect >95% of cases of 1q gain [57]. Studies demonstrating the prognostic significance of genetic markers in WT largely relied on the assumption that one sample is sufficient for detecting the marker of interest. In light of these new data, the currently accepted markers of poor prognosis must be verified in studies applying more rigorous tumor sampling. Further, multiple samples of a tumor specimen should be assessed clinically to determine risk stratification.

The ability to risk stratify WT based on patient and tumor characteristics has resulted in significant progress in improving survival while reducing exposure to toxic therapies. At the same time, these stratifications have created multiple subgroups of patients with numbers too small to power clinical trials. Thus, there is growing need for collaboration among institutions, nationally and internationally [58].

## Figures and Tables

**Table 1:** Wilms tumor stages according to the Children’s Oncology Group.

Stage	Criteria
Stage I	Confined to kidney
	Complete excision with renal capsule intact and negative resection margins
	Lymph nodes negative for Wilms tumor spread
Stage II	Regional extension beyond kidney capsule, but confined to flank
	May include:
	Tumor penetration through capsule but confined to Gerota’s fascia
	Infiltration into renal vein
	Complete excision with negative resection margins
	Lymph nodes negative for Wilms tumor spread
Stage III	Residual tumor, but confined to abdomen
	May include:
	Regional lymph node involvement
	Peritoneal contamination:
	Biopsy
	Pre- or intraoperative tumor rupture
	Tumor growth through peritoneal surface
	Positive resection margins
Stage IV	Distant metastases: Lung, liver, bone, brain
Stage V	Involvement of bilateral kidneys at diagnosis

Adapted from Davidoff (2012) [4].

**Table 2:** Current risk stratification, treatment regimens, and outcomes.

Risk	Patient/Tumor Characteristics	Current Therapy	Results	Citation
Very low	Stage I FHWT	Nephrectomy only	4-yr EFS: 89.7% (84.1–95.2%)	[48]
	Age <2 YO		4-yr OS: 100%	
	Tumor weight <550g			
Low	Stage I or II FHWT − LOH 1p/16q	Nephrectomy Regimen EE-4A	4-yr EFS: 91.2% (CI not provided) 4-yr OS: 98.4% (CI not provided)	[18]
Standard	Stage I or II FHWT	Nephrectomy	4-yr EFS: 83.9% (64.9–93.1%)	[49]
	+ LOH 1p/16q	Regimen DD-4A	4-yr OS: Not published	
	Stage III FHWT	Nephrectomy	4-yr EFS: 88% (85–91%)	[50]
	− LOH 1p/16q	Regimen DD-4A	4-yr OS: 97% (95–99%)	
		RT tumor bed + involved sites		
	Stage IV FHWT	Nephrectomy	4-yr EFS: 79.5% (71.2–87.8%)	[51]
	− LOH 1p/16q	Regimen DD-4A	4-yr OS: 96.1% (92.1–100%)	
	Isolated lung mets, RCR	RT tumor bed		
Higher	Stage IV FHWT	Nephrectomy	4-yr EFS: 88.5% (81.8–95.3%)	[51]
	− LOH 1p/16q	Regimen M	4-yr OS: 95.4% (90.9–99.8%)	
	Isolated lung mets, SIR	RT tumor bed + involved sites		
	Stage IV FHWT		Not published	
	Extrapulmonary mets			
	Stage III or IV FHWT		4-yr EFS: 91.5% (78.5–96.8%)	[49]
	+ LOH 1p/16q		4-yr OS: Not published	
High	Any DAWT	Nephrectomy	3-yr EFS: 69% (56–80%)	[52]
		Regimen UH-1	3-yr OS: Not published	
		RT tumor bed + involved sites		
	Stage IV DAWT	Nephrectomy	4-yr EFS: 57% (28–78%)	[52]
		Irinotecan/Vincristine window	4-yr OS: Not published	
		Regimen UH-2		
		RT tumor bed involved sites		
Bilateral, Multicentric, Predisposed	Bilateral WT	Induction with Regimen VAD	4-yr EFS: 82.1% (73.5–90.8%)	[53]
		(Partial) nephrectomy	4-yr OS: 94.9% (90.1–99.7%)	
		Adjuvant therapy depends on path		
	Unilateral tumors bilaterally predisposed	Induction with Regimen VA	Not published	
		(Partial) nephrectomy		
		Adjuvant therapy depends on path		
	DHPLN	Regimen VA	Not published	

WT, Wilms tumor; FHWT, favorable histology Wilms tumor; DAWT, diffuse anaplastic Wilms tumor; LOH, loss of heterozygosity; EFS, event-free survival; OS, overall survival; RT, radiation therapy; RCR, rapid complete response; SIR, slow incomplete response; DHPLN, diffuse hyperplastic perilobar nephroblastomatosis; REGIMEN EE-4A = vincristine and dactinomycin × 16 weeks; REGIMEN DD-4A = dactinomycin, vincristine, and doxorubicin × 24 weeks; REGIMEN M = vincristine, dactinomycin, doxorubicin, cylophosphamide, etoposide × 24 weeks; REGIMEN UH-1 = alternating cyclophosphamide/carboplatin/etoposide and vincristine/doxorubicin/cyclophosphamide × 30 weeks; WINDOW THERAPY = vincristine and irinotecan × 6 weeks (assessment of response at 3 weeks); REGIMEN UH-2 = cyclophosphamide/carboplatin/etoposide and vincristine/doxorubicin/cyclophosphamide + irinotecan; REGIMEN VAD = vincristine, dactinomycin, doxorubicin; REGIMEN VA = vincristine, dactinomycin.
